# Effect of monosodium glutamate on growth performance and blood biochemical parameters of rainbow trout (*Oncorhynchus mykiss* W.)

**DOI:** 10.14202/vetworld.2019.1008-1012

**Published:** 2019-07-11

**Authors:** Georgi Zhelyazkov, Deyan Stratev

**Affiliations:** 1 Department of Biology and Aquaculture, Faculty of Agriculture, Trakia University, 6000 Stara Zagora, Bulgaria; 2 Department of Food Hygiene and Control, Veterinary Legislation and Management, Faculty of Veterinary Medicine, Trakia University, 6000 Stara Zagora, Bulgaria

**Keywords:** blood biochemical parameters, growth performance, monosodium glutamate, rainbow trout

## Abstract

**Aim:**

This study aimed to evaluate the effect of dietary monosodium glutamate (MSG) on growth performance and blood biochemical parameters of rainbow trout (*Oncorhynchus mykiss* W.).

**Materials and Methods:**

A total of 200 trouts were allotted in five experimental groups (n=40) that for 60 days received feed supplemented with MSG at the following levels: 0% (K) or 0.5% (E) or 1% (F) or 3% (G) or 5% (H).

**Results:**

The average initial weight of trouts was 116.68±1.51 g (K), 116.58±1.33 g (E), 116.43±1.39 g (F), 117.40±1.47 g (G), and 115.95±1.88 g (H). At the end of the trial, control fish (K) had the lowest live weight (242.90±3.87 g) compared to Groups E (252.70±5.69 g), F (250.93±4.82 g), G (248.25±4.98 g), and H (247.95±4.74 g). Feed conversion ratio (FCR) of control group (K) was higher (1.11±0.02) versus FCR values established in Groups E (1.03±0.01), F (1.04±0.02), G (1.07±0.03), and H (1.06±0.02). Albumin, aspartate aminotransferase, gamma-glutamyl transpeptidase, alkaline phosphatase, phosphorus, magnesium, and triglyceride exhibited statistically significant differences versus controls.

**Conclusion:**

The results from the study showed that dietary supplementation of rainbow trouts with MSG increased live weight and reduced FCR. The optimum level of MSG recommended for addition to feed was 0.5%.

## Introduction

In 2016, fish production attained 171 million tonnes, 47% of which are from aquaculture. The first sale value for aquaculture in 2016 amounted to USD 232 billion. At a global scale, fish consumption also has increased – from 9 kg per capita in 1961 to 20.2 kg per capita in 2015, for example, an annual increment of 1.5% [1]. Rainbow trout (*Oncorhynchus mykiss* W.) has a worldwide distribution (apart from Antarctica) for recreational fishing and aquaculture, as it is resistant, easily reproduced; it grows rapidly and tolerates large environmental variations. Its production increased dramatically during the 1950s, especially in Europe [2,3], and in 2016, trout production share is 2% of the global aquaculture production [1].

Monosodium glutamate (MSG) is a non-essential amino acid found in all protein feeds, representing sodium salt of glutamic acid [4]. Natural foods contain free and bound MSG, and in some foods, free MSG is found in large amounts [5]. MSG is widely used as feed additive for enhancing taste. It could be suggested that its presence in foods would increase their consumption [6,7]. The U.S. Food and Drug Administration considers MSG as safe, while the European Union’s Food Safety Authority determines an intake of 30 mg/kg body weight per day as safe [7].

The use of supplements in fish diets is beneficial. Some amino acids could be used as supplements. They act as regulators of metabolism and are necessary for maintenance, growth, reproduction, and immune response. Glutamate is one of these amino acids [8]. It acts directly either after its conversion into other amino acids or in the intracellular metabolism as source of energy for enterocytes and nucleic acids precursor [9,10].

This study aimed to evaluate the effect of dietary MSG on growth performance and blood biochemical parameters of rainbow trout (*O. mykiss* W.).

## Materials and Methods

### Ethical approval

All applicable international, national, and/or institutional guidelines for the care and use of animals were followed by the authors.

### Experimental design

A total of 200 rainbow trouts were allotted in five experimental groups (n=40). The fish were kept in concrete tanks with a 0.8 m^3^ effective water volume, element of the recirculation system. Rainbow trouts were fed extruded feed “Ultra” (Alltech Coppens – The Netherlands), with 3 mm pellet size. MSG crystals were dissolved in distilled water and sprayed onto the feed of the fish from the experimental groups at either 0.5% (E), 1% (F), 3% (G), or 5% (H). All supplemented diets were prepared 1 h before feeding to complete absorption of the sprayed MSG and avoidance of its loss in the water. The feed of control trouts (K) did not contain MSG. The nutritional content of feed of the different variants is presented in Table-1. Feed was offered thrice per day at an amount equal to 3% of biomass according to the manufacturer’s instructions. The duration of the experimental period was 60 days.

**Table 1:** Nutrient content in extruded feed for rainbow trouts (*Oncorhynchus mykiss* W.).

Item	Groups				

	K	E	F	G	H
Crude protein, %	44.00	44.00	44.00	44.00	44.00
Crude lipids, %	26.00	26.00	26.00	26.00	26.00
Crude fiber, %	1.60	1.60	1.60	1.60	1.60
Crude ash, %	7.40	7.40	7.40	7.40	7.40
p-value, %	1.20	1.20	1.20	1.20	1.20
Monosodium glutamate, %	-	0.5	1	3	5
ME, MJ/kg	23.20	23.20	23.20	23.20	23.20

*1 kg feed contains: Vitamin A–10,000 IE; Vitamin D– 1240 IE; Vitamin E–200 mg; Vitamin C–250 mg. **1 kg feed contains: Fe–62 mg; Mn–26 mg; Cu–5 mg; Zn–103 mg; I–2.6 mg; Se–0.3 mg.

### Determination of growth performance

MSG influence on the weight gain and feed conversion ratio (FCR, FCR=Feed intake/weight gain) of rainbow trouts in the recirculation system was assessed by measuring individual fish weight (g) at the 30^th^ day and at the end of the experiment. At the end of the experimental period, the survival rate (%), weight gain (g), and FCR were also calculated.

### Determination of blood biochemical parameters

Blood samples were obtained on the last day of the experimental period through caudal vessels puncture using EDTA containers. Glucose, urea, creatinine, total protein, albumin (ALB), aspartate aminotransferase (ASAT), alanine aminotransferase, gamma-glutamyl transpeptidase (GGT), alkaline phosphatase (ALP), calcium, phosphorus (P), magnesium (Mg), triglyceride (TG), and cholesterol concentrations were determined using a BS-120 Chemistry Analyzer (Mindray, China).

### Economic analysis

Economic efficiency of dietary MSG supplementation in rainbow trouts reared in a recirculation system was assessed by estimating the cost of feed per kg of weight gain and by calculating the economic conversion ratio (ECR). ECR was calculated according to Piedecausa *et al*. [11]: ECR = Cost of diet×FCR.

### Hydrochemical analysis

Water temperature, dissolved oxygen, pH, and electrical conductivity were determined using MultiLine P4 (Xylem Analytics Germany Sales GmbH & Co. KG, WTW). Nitrates and nitrites were determined by spectrophotometric techniques according to Bulgarian State Standards (BSS) 17.1.4.12:1979 and BSS ISO 26777:1997, respectively. The water parameters were measured every day during the experimental period.

### Statistical analysis

Data were presented as means±standard deviation and possible differences were detected by t-test using Statistica^®^ Version 6.0 (StatSoft, Tulsa, OK, USA). The statistical significance was determined at p<0.05.

## Results and Discussion

During the experimental period, water parameters in the recirculation system were maintained within optimum ranges for rainbow trout farming (Table-2).

**Table 2:** Water parameters in the recirculation system for rainbow trout (*Oncorhynchus mykiss* W.) farming.

Parameters	n	x̄±SD
Temperature, °C	60	13.90±1.30
Dissolved oxygen, mg/l	60	9.86±0.54
pH	60	7.64±0.63
Nitrates, mg/l	60	0.25±0.05
Nitrites, mg/l	60	0.006±0.001
Electric conductivity, μs/cm	60	530.00±6.78

### Growth performance of rainbow trout (*O. mykiss* W.)

Growth performance parameters in experimental fish are presented in Table-3. The average initial weight of supplemented fish was 116.68±1.51 g (Group K), 116.58±1.33 g (Group E), 116.43±1.39 g (Group F), 117.40±1.47 g (Group G), and 115.95±1.88 g (Group H) without statistically significant intergroup differences (p>0.05). In the middle of the experimental period, fish from Groups E (180.12±4.16 g) and F (180.24±4.68 g) were heavier than those from control group (K) (174.50±4.50 g) (p<0.05), while trouts from Groups G (172.94±3.90 g) and H (173.25±4.49 g) weighed less than controls (p<0.05). At the end of the trial, control trouts had the lowest average live weight (242.90±3.87 g) compared to fish from Group E (252.70±5.69 g), F (250.93±4.82 g), G (248.25±4.98 g), and H (247.95±4.74 g). The differences were significant (p<0.05) between controls (К) and Groups E, F, G, and insignificant (p>0.05) versus Group H. At the study’s end, the average individual live weight of fish from Group E (136.12±2.55 g), F (134.50±3.02 g), G (130.85±2.46 g), and H (132.00±2.18 g) exceeded that of control trouts (126.22±2.32 g) (p<0.05).

**Table 3:** Growth performance parameters of rainbow trouts (*Oncorhynchus mykiss* W.).

Parameter	n	K	E	F	G	H	Significance
				
		x̄±SD	x̄±SD	x̄±SD	x̄±SD	x̄±SD	
Initial body weight, g	40	116.68±1.51^a^	116.58±1.33^a^	116.43±1.39^a^	117.40±1.47^a^	115.95±1.88^a^	NS
Body weight in the middle of the trial, g	40	174.50±4.50^a^	180.12±4.16^b^	180.24±4.68^c^	172.94±3.90^a^	173.25±4.49^a^	*
Final body weight, g	40	242.90±3.87^a^	252.70±5.69^b^	250.93±4.82^c^	248.25±4.98^d^	247.95±4.74^a^	*
Survival rate, %		100	100	100	100	100	
Average individual weight gain, g	40	126.22±2.32^a^	136.12±2.55^b^	134.50±3.02^c^	130.85±2.46^d^	132.00±2.18^e^	*
FCR	40	1.11±0.02^a^	1.03±0.01^b^	1.04±0.02^c^	1.07±0.03^d^	1.06±0.02^e^	*

Values indicated with different superscripts are significantly different;

* p<0.05, NS=Non-significant, FCR=Feed conversion ratio

FCR of controls (К) (1.11±0.02) was higher compared to that of Group E (1.03±0.01), F (1.04±0.02), G (1.07±0.03), and H (1.06±0.02) (p<0.05). The cost of extruded feed for rainbow trouts was 1500.00 €/tonne (VAT excluded). MSG was dispersed on pellets of experimental fish, which increased the cost of feed. The increase for groups that received 0.5%, 1%, 3%, and 5% MSG was 10.50 €/tonne, 21.00 €/tonne, 63.00 €/tonne, and 105.00 €/tonne. The ECR was the best for trouts from Group E supplemented with 0.5% MSG (1.556) compared to control group (K) (1.665) and other groups F (1.582), G (1.672), and H (1.701) (Table-4). In intensive aquaculture systems [12], feed costs range from 30% to 60% of operative costs meaning that feeding management is especially important. The specific growth rate and FCR are the two primary factors demonstrating the efficiency of feeding management and economic efficiency of aquaculture. Our study showed that the addition of MSG to feed of trouts reduced FCR, for example, one unit of weight gain was achieved with lower amount of feed.

**Table 4:** Economic efficiency of dietary monosodium glutamate supplementation.

Item	K	E	F	G	H
Price, €/t feed(VAT excluded)	1500.00	1510.50	1521.00	1563.00	1605.00
ECR*	1.665	1.556	1.582	1.672	1.701

* The lowest value shows the best ECR. ECR=Economic conversion ratio

The addition of MSG to extruded pellets for rainbow trouts increased the live weight and did not have any negative effect on survival of supplemented fish. According to Oehme *et al*. [8], glutamate acts as attractant for fish and its addition to fish diet increases its intake due to both olfactory and taste stimuli. Yan and Qiu-Zhou [9] proved that the addition of 0.4%, 0.8%, and 1.2% glutamate to feed of juvenile Jian carp (*Cyprinus carpio* var. Jian) resulted in higher live weight, improved feed conversion, increased body length and intestinal weight, and increased intestinal enzymatic activity. According to the authors, these beneficial effects were due to glutamate, which is a precursor of nucleotide biosynthesis in rapidly dividing cells and source of energy for enterocytes. Yoshida *et al*. [10] proved that glutamate had a positive effect on growth performance of rainbow trouts. Supplementation of soybean meal-based diet with 2% glutamate for 8 weeks resulted in improved growth through reformation of proximal intestinal microvilli. The authors assumed that the utilization of dietary glutamate possibly played an important role in intestinal and systemic metabolism. Yan and Qiu-Zhou [9] recommended supplementation of feed of fish with glutamate for prevention of intestinal epithelium damage. Moreover, in other farm animals, the dietary supplementation of 1% glutamate resulted in improved growth and stimulation of gastrointestinal tract development of broilers, although higher levels had an adverse effect [13]. Rezaei *et al*. [14] provided evidence that MSG levels up to 4% were safe and improved the growth of weaned pigs.

### Blood biochemical parameters of rainbow trout (*O. mykiss* W.)

Seven of 14 tested biochemical blood parameters exhibited statistically significant differences (Table-5). Blood ALB in control group (К) (27.83±1.27 g/l) was considerably different versus Group E (25.10±1.80 g/l), F (23.93±2.80 g/l), and H (23.10±2.38 g/l). According to Tawfik and Al-Badr [15], decreased concentration of blood ALB in MSG-treated adult rats at a concentration of 1.6 mg/g body weight was a sign of diminished liver synthetic function. Substantial differences between controls (64.75±58.02 U/l) and Group G (175.50±67.42 U/l) were established for blood ASAT concentrations. In Groups К, E, and F, GGT was not found out, while its levels in Group G and H were 2.25±1.71 U/l and 3.50±3.87 U/l, respectively. Elevated GGT serum concentration in MSG-treated adult rats at a concentration of 1.6 mg/g body weight indicated liver damage by oxidative stress [15]. ALP concentrations in control group (132.50±96.41 U/l) were significantly lower compared to Groups G (455.25±197.21 U/l) and H (583.00±205.67 U/l). Al-Mousawi [16] fed adult albino rats with diet supplemented with 5 g/kg MSG for 30 days and found out elevated serum concentrations of AST, ALT, and ALP due to damage of hepatocytes and release of these enzymes into bloodstream. Significant differences were demonstrated for phosphorus concentrations in controls (К) (3.45±0.27 mmol/l) versus Group H (4.35±0.55 mmol/l). Magnesium in blood in control trouts (1.37±0.08 mmol/l) exceeded levels in Group F (0.57±0.56 mmol/l). TG concentrations in non-supplemented fish (1.60±0.21 mmol/l) were significantly higher than those in Group G (1.19±0.21 mmol/l) and H (1.08±0.03 mmol/l). Contrary to our findings, Ahluwalia and Malik [17] proved that subcutaneous administration of 4 and 8 mg/g MSG in adult male mice resulted in increased serum concentrations of total lipids, phospholipids, TGs, and free fatty acids representing major changes in obesity. According to Coşkun *et al*. [2] and Fazio *et al*. [3], blood biochemical parameters in fish were easily influenced by water quality and temperature, blood collection technique, feeding, sexual maturity, and photoperiod. To the best of our knowledge, no data are available for MSG effects on blood biochemistry of rainbow trout (*O. mykiss* W.).

**Table 5:** Blood biochemical parameters of rainbow trouts (*Oncorhynchus mykiss* W.).

Parameters	n	K	E	F	G	H	Significance
				
		x̄±SD	x̄±SD	x̄±SD	x̄±SD	x̄±SD	
GLU, mmol/l	4	9.63±2.08^a^	8.37±1.17^a^	9.61±3.54^a^	11.06±3.32^a^	12.06±2.52^a^	NS
UREA, mmol/l	4	0.73±0.21^a^	0.85±0.13^a^	0.63±0.05^a^	0.78±0.10^a^	0.63±0.15^a^	NS
CREA, μmol/l	4	16.50±1.29^a^	16.00±3.16^a^	16.50±0.58^a^	17.00±1.41^a^	17.25±3.59^a^	NS
TP, g/l	4	51.73±1.90^a^	48.00±2.39^a^	46.18±6.43^a^	52.18±5.81^a^	47.10±4.25^a^	NS
ALB, g/l	4	27.83±1.27^a^	25.10±1.80^b^	23.93±2.80^c^	25.33±2.54^a^	23.10±2.38^d^	*
ASAT, U/l	4	64.75±58.02^a^	46.25±46.95^a^	45.00±46.63^a^	175.50±67.42^b^	104.50±123.22^a^	*
ALAT, U/l	4	16.25±9.74^a^	21.50±8.35^a^	18.75±13.96^a^	8.25±3.59^a^	11.25±12.31^a^	NS
GGT, U/l	4	0.00±0.00^a^	0.00±0.00^a^	0.00±0.00^a^	2.25±1.71^b^	3.50±3.87^c^	*
ALP, U/l	4	132.50±96.41^a^	223.50±88.71^a^	168.50±50.34^a^	455.25±197.21^b^	583.00±205.67^c^	**
Ca, mmol/l	4	3.34±1.45^a^	1.60±0.34^a^	1.25±1.04^a^	3.64±1.42^a^	3.50±0.45^a^	NS
P, mmol/l	4	3.45±0.27^a^	3.60±0.20^a^	3.26±0.21^a^	4.21±0.78^a^	4.35±0.55^b^	*
Mg, mmol/l	4	1.37±0.08^a^	0.91±0.58^a^	0.57±0.56^b^	0.84±0.44^a^	1.14±0.21^a^	*
TG, mmol/l	4	1.60±0.21^a^	1.52±0.06^a^	1.56±0.07^a^	1.19±0.21^b^	1.08±0.03^c^	**
CHOL, mmol/l	4	8.63±1.16^a^	8.33±0.74^a^	7.98±1.61^a^	8.23±1.98^a^	9.20±1.00^a^	NS

Values indicated with different superscripts are significantly different;

* p<0.05,

** p<0.01, NS=Non-significant, GLU=Glucose, CREA=Creatinine, TP=Total protein, ALB=Albumin, ASAT=Aspartate aminotransferase, ALAT=Alanine aminotransferase, GGT=Gamma-glutamyl transpeptidase, ALP=Alkaline phosphatase, Ca=Calcium, P=Phosphorus, Mg=Magnesium, TG=Triglyceride, CHOL=Cholesterol

## Conclusion

The results from the study showed that dietary supplementation of rainbow trouts with MSG increased live weight and reduced FCR. The MSG supplement influenced blood concentrations of ALB, ASAT, GGT, ALP, P, Mg, and TG and had no adverse effect on survival rates. The optimum level of MSG recommended for addition to feed on the basis of ECR was 0.5%.

## Authors’ Contributions

GZ and DS contributed equally to study designing, experimental work, data analysis, and manuscript writing. Both authors read and approved the final manuscript.

## References

[1] Food and Agriculture Organization. The State of World Fisheries and Aquaculture 2018. Meeting the Sustainable Development Goals

[2] Coşkun O.F, Aydın D, Duman F (2016). Comparison of some blood parameters of rainbow trout (*Oncorhynchus mykiss*) living in running and still water, Iran. J. Fish. Sci.

[3] Fazio F, Saoca C, Piccione G, Kesbiç O.S, Acar U (2016). Comparative study of some hematological and biochemical parameters of Italian and Turkish farmed rainbow trout *Oncorhynchus mykiss* (Walbaum 1792). Turk. J. Fish. Aquat. Sci.

[4] Henry-Unaeze H.N (2017). Update on food safety of monosodium L-glutamate (MSG). Pathophysiology.

[5] Shi Z, Taylor A.W, Yuan B, Zuo H, Wittert G.A (2014). Monosodium glutamate intake is inversely related to the risk of hyperglycemia. Clin. Nutr.

[6] Masic U, Yeomans M.R (2013). Does monosodium glutamate interact with macronutrient composition to influence subsequent appetite?Physiol. Behav.

[7] Peng Q, Huo D, Ma C, Jiang S, Wang L, Zhang J (2018). Monosodium glutamate induces limited modulation in gut microbiota. J. Funct. Foods.

[8] Oehme M, Grammes F, Takle H, Zambonino-Infante J.L, Refstie S, Thomassen M.S, Kjell-Arne R, Terjesen B.F (2010). Dietary supplementation of glutamate and arginine to Atlantic salmon (*Salmo salar* L.). increases growth during the first autumn in sea. Aquaculture.

[9] Yan L, Qiu-Zhou X (2006). Dietary glutamine supplementation improves structure and function of intestine of juvenile Jian carp (*Cyprinus carpio* var. Jian). Aquaculture.

[10] Yoshida C, Maekawa M, Bannai M, Yamamoto T (2016). Glutamate promotes nucleotide synthesis in the gut and improves availability of soybean meal feed in rainbow trout. SpringerPlus.

[11] Piedecausa M.A, Mazón M.J, García B.G, Hernández M.D (2007). Effects of total replacement of fish oil by vegetable oils in the diets of sharpsnout seabream (*Diplodus puntazzo*). Aquaculture.

[12] Rad F, Köksal G, Kindir M (2003). Growth performance and food conversion ratio of Siberian sturgeon (*Acipenser baeri* Brandt) at different daily feeding rates. Turk. J. Vet. Anim. Sci.

[13] Soltan M.A (2009). Influence of dietary glutamine supplementation on growth performance, small intestinal morphology, immune response and some blood parameters of broiler chickens. Int. J. Poult. Sci.

[14] Rezaei R, Knabe D.A, Tekwe C.D, Dahanayaka S, Ficken M.D, Fielder S.E, Eide S.J, Lovering S.L, Wu G (2013). Dietary supplementation with monosodium glutamate is safe and improves growth performance in postweaning pigs. Amino Acids.

[15] Tawfik M.S, Badr Al-N (2012). Adverse effects of monosodium glutamate on liver and kidney functions in adult rats and potential protective effect of Vitamins C and E. Food Nutr. Sci.

[16] Al-Mousawi N.H (2017). Study on effect of glutamate monosodium exposure on some blood and biochemical parameters in adult albino rats. J. Entomol. Zool. Stud.

[17] Ahluwalia P, Malik V.B.T (1989). Effects of monosodium glutamate (MSG) on serum lipids, blood glucose and cholesterol in adult male mice. Toxicol. Lett.

